# Dr. Edward Everett Stanbro

**Published:** 1899-12

**Authors:** 


### OBITUARY.

DR. EDWARD EVERETT STANBRO, U. of B.,'92, died at his home in Buffalo, November 5, 1899, of phthisis pulmonalis, aged 30 years. Dr. Stanbro was well known to the younger generation of medical men in the city. Although on account of ill-health he was unable to establish himself as a physician, yet such were his life and character, that the medical profession is honored in being able to claim him as one of its members.

Edward Everett Stanbro was born in Springville, Erie County, N. Y., in 1869. He was the second son of a physician well-known in his time, Dr. George G. Stanbro. He was educated in the Academy of Springville and later entered the University of Buffalo, from which institution he received first the degree in pharmacy, later that in medicine. Shortly after his graduation in medicine, in 1892, he was obliged to go to Colorado, the double work which he had done in college having undermined a constitution never too strong. Returning in 1893, he practised medicine for a short time and later pharmacy. A second failure in health obliged him to change his occupation, and in 1897 he became a member of the staff of the *Buffalo Courier*. At the time of his death he held the position of dramatic editor on that paper, having won promotion by creditable and earnest work.

Dr. Stanbro was a modest man. Had he been asked he would have pronounced his life a failure. Measured by what he wished to do and was capable of accomplishing, but for the limitations placed about him, it was. But if we estimate the value of his life by the qualities which go to make true greatness it was a notable success. Those who knew his brave battle with disease and death, those who were cheered by his cheerfulness and gladdened by his mirth, who knew that thoughtfulness for others which showed itself in courtesy to all, in kindliness and helpfulness to those whom he called friend, feel that life is forever richer because such an one has lived. Unassuming, kindly, sympathetic, most of all courageous,

"He bore without abuse
The grand old name of gentleman."

